# Genotyping Based on CRISPR Loci Diversity and Pathogenic Potential of Diarrheagenic *Escherichia coli*

**DOI:** 10.3389/fmicb.2022.852662

**Published:** 2022-03-02

**Authors:** Zhiye Bai, Shiqin Zhang, Xiang Wang, Muhammad Zohaib Aslam, Wen Wang, Hongmei Li, Qingli Dong

**Affiliations:** ^1^School of Health Science and Engineering, University of Shanghai for Science and Technology, Shanghai, China; ^2^State Key Laboratory for Managing Biotic and Chemical Threats to the Quality and Safety of Agro-products, MOA Laboratory of Quality and Safety Risk Assessment for Agro-products (Hangzhou), Institute of Agro-product Safety and Nutrition, Zhejiang Academy of Agricultural Sciences, Hangzhou, China

**Keywords:** diarrheagenic *Escherichia coli*, CRISPR typing, serotyping, virulence genes, Caco-2 cell

## Abstract

Diarrheagenic *Escherichia coli* (DEC) can cause epidemic diarrhea worldwide. The pathogenic potential of different strains is diverse and the continuous emergence of pathogenic strains has brought serious harm to public health. Accurately distinguishing and identifying DEC with different virulence is necessary for epidemiological surveillance and investigation. Clustered regularly interspaced short palindromic repeats (CRISPR) typing is a new molecular method that can distinguish pathogenic bacteria excellently and has shown great promise in DEC typing. The purpose of this study was to investigate the discrimination of CRISPR typing method for DEC and explore the pathogenicity potential of DEC based on CRISPR types (CT). The whole genome sequences of 789 DEC strains downloaded from the database were applied CRISPR typing and serotyping. The D value (Simpson’s index) with 0.9709 determined that CRISPR typing had a higher discrimination. Moreover, the same H antigen strains with different O seemed to share more identical spacers. Further analyzing the strains CRISPR types and the number of virulence genes, it was found that there was a significant correlation between the CRISPR types and the number of virulence genes (*p* < 0.01). The strains with the largest number of virulence genes concentrated in CT25 and CT56 and the number of virulence genes in CT264 was the least, indicating that the pathway potential of different CRISPR types was variable. Combined with the Caco-2 cell assay of the laboratory strains, the invasion capacity of STEC strains of different CRISPR types was different and there was no significant difference in the invasion rate between different CRISPR type strains (*p* > 0.05). In the future, with the increase of the number of strains that can be studied experimentally, the relationship between CRISPR types and adhesion and invasion capacities will be further clarified.

## Introduction

Diarrhea illness is one of the global healthcare problems. Diarrheagenic *Escherichia coli* (DEC) is an important agent of epidemic diarrhea worldwide especially in some underdeveloped areas with poor sanitation among bacterial pathogens (Gomes et al., 2016). DEC can be divided into five categories according to its virulence characteristics: enterotoxigenic *E. coli* (ETEC), enteroaggregative *E. coli* (EAEC), enteroinvasive *E. coli* (EIEC), enteropathogenic *E. coli* (EPEC), and Shiga toxin–producing *E. coli* (STEC) (Shahbazi et al., 2021). Strains can be identified according to the characteristic virulence genes of different DEC categories: *stx1*, *stx2*, and *eae* in STEC; *aggR* and *astA* in EAEC; *invE* and *ipaH* in EIEC; *est* and *elt* in ETEC. Furthermore, typical (*eae*^+^, *bfp*^+^) EPEC and atypical (*eae*^+^, *bfp*^–^, *stx1*^–^, *stx2*^–^) EPEC can be distinguished (Fujioka et al., 2013; Rodrigues et al., 2016; Spano et al., 2021). Some studies have shown the association between EPEC and diarrhea. Moreover, EPEC was the main cause of death in children with diarrhea in developing countries (Scaletsky et al., 2002; Afset et al., 2004; Hernandes et al., 2009). ETEC was the most common cause of travelers’ diarrhea followed by EAEC in developed and developing countries (Daniels, 2005; Croxen and Finlay, 2010). Large outbreaks of diarrheal diseases were caused by EIEC and proved to be indeed associated with diarrhea (Manuel et al., 1992). STEC was first associated with hemolytic uremic syndrome (HUS) and hemorrhagic colitis (HC) in the 1980s, and subsequently with uncomplicated diarrhea (Karmali et al., 1983; Riley et al., 1983; Pai et al., 1988). Both *stx1* and *stx2* carried by STEC are the most common in HUS patients. The *stx* were proved to reach endothelial cells and induce cascade thrombosis and inflammatory changes, leading to HUS in kidney and other organs (Keir et al., 2012). Both *eae* and *ehxA* carried by STEC also can cause serious diseases in humans (Shen et al., 2015). STEC can lead to typical colonic attachment and elimination of lesions by adhesion to intestinal epithelial cells, which can cause diarrhea or bloody diarrhea (Bruyand et al., 2018). This feature is primarily related to the intima of a protein encoded by the *eae* carried by the chromosomal locus of enterocyte effacement (LEE). Ruminants such as cattle and sheep are the main hosts of STEC. STEC infections are mainly foodborne, and from bovine feces and water contaminated by animal feces (Boerlin et al., 1999; Muniesa et al., 2006).

Different *E. coli* pathogenic categories have different characteristic virulence genes, and virulence genes can be transferred horizontally in pathogenic *E. coli*, allowing pathogenic strains to acquire new biological characteristics and even lead to the generation of new strains (Kyle et al., 2012). Horizontal gene transfer (HGT) between bacteria by Mobile Genetic Elements (MGE) carrying virulence genes has resulted in the continuous emergence of highly virulent strains, which has brought serious harm to public health (Xiong et al., 2012; Yan et al., 2015). Therefore, it is an important task for epidemiological surveillance and investigation to accurately distinguish and identify diarrheagenic *E. coli* with different virulence. Serotypes can be used as a characteristic indicator of bacteria and long term for DEC typing in view of most of the pathogenic strains contained in the known O:H serotypes. For example, O157:H7 caused severe disease harming to patients. In addition, some studies have shown that O26:H11, O45:H2, O103:H2, O111:H8(NM), O121:H19, and O145:NM are also the main serotypes of major foodborne diseases (Karmali, 2018; Spano et al., 2021). Consequently, O:H serotyping plays an important role in bacterial classification, epidemiological monitoring, epidemic detection, and other aspects, and it can also provide information directly related to antigen response (Joensen et al., 2015). However, serotyping still has limitations, so it is necessary to use new typing methods to compensate for the shortcomings of isolates serotyping.

Clustered regularly interspaced short palindromic repeats (CRISPR) is one of the most rapidly evolving components of the genome, mainly composed of almost identical repeats and highly specific spacers. The insertion and deletion of spacers in CRISPR arrays reflect the polymorphism of bacterial evolution (Deveau et al., 2010; Makarova et al., 2011). CRISPR and CRISPR-associated sequence (CAS) proteins together form the CRISPR/CAS system (Díez-Villaseñor et al., 2010; Toro et al., 2014), which can acquire immunity for prokaryotes through invading phages and plasmids, thereby resisting phage infection and limiting the HGT (Marraffini and Sontheimer, 2008, 2010). Some researchers found that spacers are derived from foreign genetic elements such as exogenous plasmids or phages (Bolotin et al., 2005; Mojica et al., 2005; Pourcel et al., 2005), which is related to the mechanism of action of CRISPR/CAS system. The CRISPRs of different strains are divergence and the high polymorphism of spacers can be used as high discrimination biomarkers for genotyping. Four CRISPR loci have been identified in *E. coli*, namely CRISPR1, CRISPR2, CRISPR3, and CRISPR4 (Díez-Villaseñor et al., 2010; Yin et al., 2013; Toro et al., 2014). A recent study (Long et al., 2019) classified 413 *E. coli* strains into 23 types according to CRISPR3 and CRISPR4 loci. In addition, CRISPR typing method has been applied to *Salmonella*, *Campylobacter jejuni*, *Cronobacter*, and other foodborne pathogens (Li et al., 2018; Zeng et al., 2019; Yeh and Awad, 2020), proving that CRISPR typing method has become popular. However, pathogenicity potential of DEC based on CRISPR types seldom focus on and needs further studies.

The purpose of this study was to investigate the distinguishing ability of CRISPR typing for DEC and explore the pathogenicity potential of DEC strains. We performed CRISPR typing of DEC strains and analyzed virulence genes of different CRISPR types. In addition, we combined the results of Caco-2 cell assay of laboratory *E. coli* to further explore the adhesion and invasion capacities of STEC strains based on CRISPR types.

## Materials and Methods

### Strains

#### Sequence Collection

The whole genome sequences of DEC were downloaded from the National Center for Biotechnology Information (NCBI) database (updated before May 19, 2021). The strains were classified according to the characteristic genes of DEC.

#### Laboratory Strains

Twelve STEC strains were screened in the laboratory. A total of 8 strains were isolated from ground beef and 4 strains were isolated from cattle feces. The whole genomes of the strains have been sequenced.

### Serotyping

SerotypeFinder 2.0 Web-based tool^1^ was applied to analyze the serotype of the whole genome sequence of laboratory strains and DEC downloaded by NCBI with the following parameters: 85% threshold for %ID and 60% minimum length.

### Clustered Regularly Interspaced Short Palindromic Repeats Typing

The identification of CRISPR loci and the spacers were extracted using CRISPRfinder (Grissa et al., 2007) and BLAST. The comparison of spacer sequences and obtaining unique spacers were identified by ClustalX (Larkin et al., 2007). Each unique spacer was recorded associated a single number beginning with 1. Then, every CRISPR array with multiple spacers was assigned a number as a spacer code. CRISPR typing was performed by combining CRISPR1, CRISPR2, CRISPR3, and CRISPR4 into one allele and displayed this as an arrangement of CRISPR spacers. The CRISPR type (CT) of each strain was defined using a specific number beginning with 1. The discrimination index (D) was calculated based on the Simpson’s index of diversity with the equation as previously defined (Hunter and Gaston, 1988; Yeh and Awad, 2020).

### Identification of Virulence Genes

VirulenceFinder 2.0 Web-based tool^2^ was used to determine virulence genes of DEC genomes with the following parameters: 90% threshold for %ID and 60% minimum length.

### Adherence and Invasion Assay of Caco-2 Cells

#### Cell Culture

Caco-2 cells (FH0029) were purchased from the FuHeng BioLogy (Shanghai, China). The cells were removed from the liquid nitrogen and placed in 37°C water bath, thawed by shaking. The Caco-2 cells were placed in Dulbecco’s Modified Eagle’s Medium (DMEM), supplemented with 10–20% (v/v) fetal bovine serum (FBS), 1% (v/v) non-essential amino acids (Coolaber, Beijing, China), and 1% (v/v) penicillin–streptomycin solution. Cells were grown at 37°C in a humidified atmosphere of 5% CO_2_ and 95% air conditions (Klingberg et al., 2008). For the experimental assays, Caco-2 cells were grown in 12-well tissue culture plates (Greiner Bio-One, Frickenhausen, Germany) until monolayers were developed.

#### Adhesion Assay

*In vitro*, the adhesion capacity of DEC to Caco-2 cells was studied, and DEC in this study belonged to STEC. The STEC isolates grow overnight at 37°C in Luria–Bertani (LB) culture. Bacteria suspension (1 ml) was added to each well of the tissue culture plates containing monolayers of Caco-2 cells and the precise number of bacteria in the inoculum (10^7^–10^8^ CFU/ml) added to monolayers was determined retrospectively by serial dilutions and plate counting. After 2 h of incubation at 37°C, the Caco-2 cells were washed three times with PBS to remove non-attached bacterial cells (Olesen and Jespersen, 2010). One milliliter 1% Triton X-100 (Applichem, Darmstadt, Germany) was added to each well to loosen and lyse the Caco-2 monolayers. Appropriate serial dilutions of the Caco-2/DEC mixtures were plated on LB agar plates to determine the number of adhered Caco-2 cells. For each assay, the mean value of adherent bacteria was determined and the SEM from triplicate experiments was calculated. The adhesion index was calculated as the ratio of the number of adhered cells to the amount of inoculation × 100%. Results were expressed as% bacteria adhered relative to inoculum.

#### Invasion Assay

To determine bacterial invasion, the bacteria solution was added to each well of a tissue culture plate containing Caco-2 monolayer cells, incubated at 37°C for 2 h and then washed with PBS 3 times. Then 1 ml 100 μg/L penicillin–streptomycin solution was added and incubated for 1 h to kill remaining viable extracellular bacteria. The cells were lysed with 1% Triton X-100 for 5 min after washing the penicillin–streptomycin solution and a serial dilution method was used to quantify viable intracellular bacteria. LB agar plates were used to determine the number of invasive bacteria. The mean value of invasive bacteria was determined and the SEM from triplicate experiments was calculated. Results were expressed as% of invasive bacteria relative to inoculum.

### Statistics

Results were analyzed using GraphPad Prism 8 and SPSS 25. The ANOVA analysis followed by a Duncan test at 95% confidence limits were applied to determine the differences in the STEC adhesive and invasion capacities, and the difference in the number of virulence genes among strains of different CRISPR types. *P*-values of <0.05 were considered statistically significant. Pearson test was used to analyze the correlation between CRISPR typing and virulence gene number of strains.

## Results and Discussion

### Sequence Collection

Complete genome sequences of 789 DEC strains were downloaded from NCBI database, including 521 strains of STEC, 116 strains of EAEC, 105 strains of EPEC, 30 strains of EIEC, and 17 strains of ETEC. The NCBI accession numbers of all genomes analyzed in the current work were listed in Supplementary Table 1.

### Identification of Clustered Regularly Interspaced Short Palindromic Repeats Loci

**FIGURE 1:**
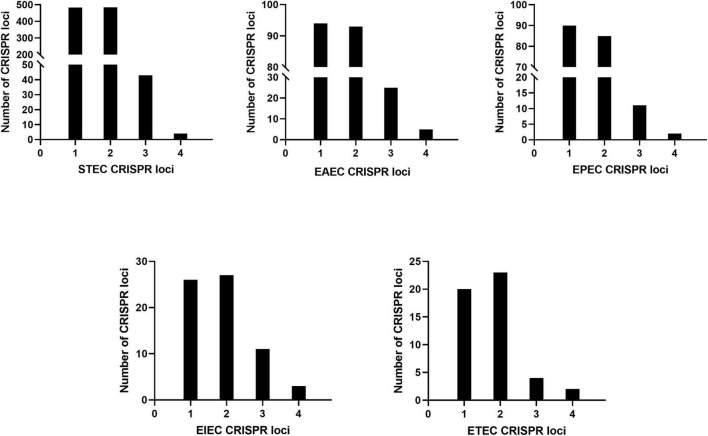
Number of DEC CRISPR loci.

The CRISPR1, CRISPR2, CRISPR3, and CRISPR4 loci of DEC were identified and extracted. A total of 733 DEC strains had CRISPR loci. The upstream and downstream sequences of CRISPR loci were relatively conserved. The CRISPR1 locus was located between *iap* and *cysH*, the CRISPR2 locus was located between *ygcE* and *ygcF*, and CRISPR3 and CRISPR4 [named CRISPR4.1 and CRISPR4.2, respectively, by Díez-Villaseñor et al. (2010)] were located between *clpA* and *infA*. The results are consistent with a previous study (Díez-Villaseñor et al., 2010). The number of CRISPR loci varies with DEC types, as shown in Figure 1.

The number of CRISPR1 and CRISPR2 loci was the largest with 713 respectively, the number of CRISPR3 loci was 94, and the number of CRISPR4 loci was 16. STEC, EAEC, EPEC, EIEC, and ETEC had more CRISPR1 and CRISPR2 loci than CRISPR3 and CRISPR4, and the number of CRISPR4 loci was the least.

### Serotyping

Among 789 DEC strains, the serotypes of 747 strains were identified. The strains were divided into 212 serotypes, of which O157:H7 accounted for a large proportion with 182 strains. The isolates of O157:H7 were mainly isolated from the United States, Netherlands, Sweden, and other places, which were associated with some food-borne disease outbreaks caused by STEC. O157:H7, O111:H8, O26:H11, O145:H28, and O121:H19 were the dominant serotypes of STEC. These serotypes most frequently implicated in outbreaks and sporadic cases of HC and HUS. The dominant serotypes of EAEC were O6:H1, O153:H30, and the dominant serotypes of EPEC were O55:H51 and O157:H16. The serotypes of EIEC and ETEC were dispersed. In addition, 119 strains had 119 serotypes, individually. The D value (Simpson’s index) for DEC strains serotyping was 0.9316.

All 12 STEC strains from our laboratory were divided into different serotypes. Among them, 4 strains isolated from cattle feces belong to O136:H12, and 3 strains isolated from ground beef were from O157:H7, O26:H11, O181:H31, O178:H19, O105:H8, and ONT:H10 distributed in single strain respectively.

### Clustered Regularly Interspaced Short Palindromic Repeats Typing

**FIGURE 2:**
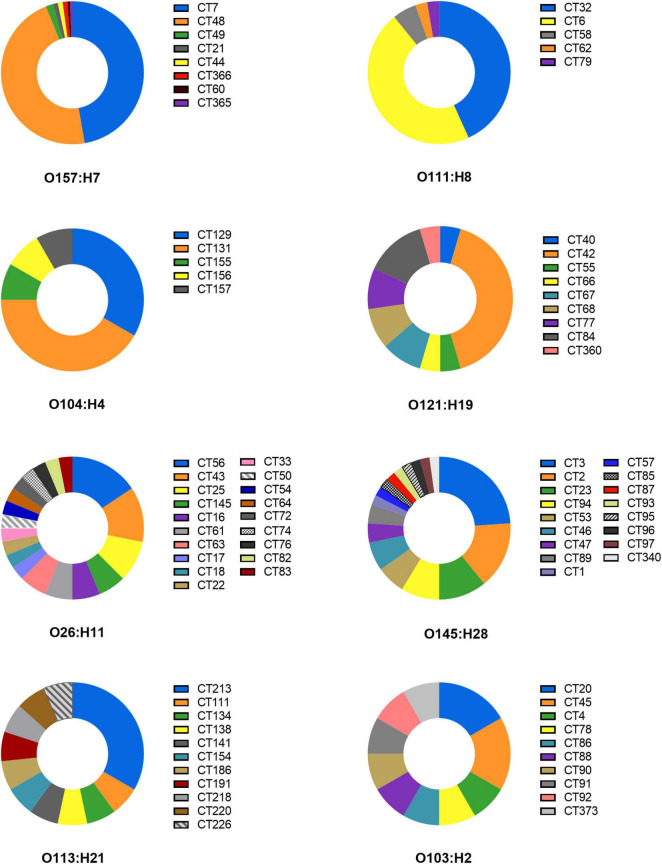
CRISPR typing distribution of the same serotype strains.

The downloaded genome sequences of DEC strains were classified by CRISPR typing method, and a total of 1,878 unique CRISPR spacers were obtained through alignment. The distributions of spacer numbers from each location seemed to be random. According to the different arrangement of these spacers to determine the CRISPR classification, the bacteria were divided into 397 CRISPR types (CT). CRISPR typing results of DEC are shown in Supplementary Table 2. CT7 (*n* = 86) was the most prevalent followed by CT48 (*n* = 85) and two kinds of CT were STEC serotype O157:H7. The strain number including CT6, CT32, and CT287 were 17, 16, and 10, respectively. The strains of other CTs were less than 10. 325 types of CRISPR typing were the least (*n* = 1). The D value (Simpson’s index) with 0.9709 determining the discriminatory power of CRISPR typing method indicated that CRISPR typing method had a higher discriminatory power than serotyping for strains, which was related to the polymorphism of CRISPR. In addition, the same serotype can be divided into different CRISPR types (Figure 2).

O157:H7 serotype was divided into 8 CRISPR types including CT7, CT21, CT44, CT48, CT49, CT60, CT365, CT366; O111:H8 contained CT6, CT32, CT58, CT62, and CT79; O104:H4 was separated by CT129, CT131, CT155, CT156, and CT157; O121:H19 included CT40, CT42, CT55, CT66, CT67, CT68, CT77, CT84, and CT360. Otherwise, O26:H11, O145:H28, O113:H21, and O103:H2 contained more than 10 CRISPR types. CRISPR typing method had a higher ability to distinguish strains than serotypes. The study suggests the possibility of serotype prompting through CRISPR arrays. It is worth mentioning that the same H antigen strains with different O seemed to share more identical spacers, while the same O group with different H could not share spacers or had fewer spacers in CRISPR. For example, O26:H11 and O116:H11 had the same CRISPR typing (CT145), which means that spacers were arranged exactly the same. O111:H11 and O69:H11 had 6 identical spacers in CRRSPR1 and completely consistent spacers in CRISPR2, while O26:H11 and O26:H36 had only one common spacer (Supplementary Table 2). Similar results in the study of STEC were found by another study (Toro et al., 2014), indicating that H antigen loci are more evolutionarily stable than O antigen because of the diversity of O antigen due to the O antigen transformation caused by gene level transfer (Rump et al., 2010; Wang et al., 2012).

**FIGURE 3:**
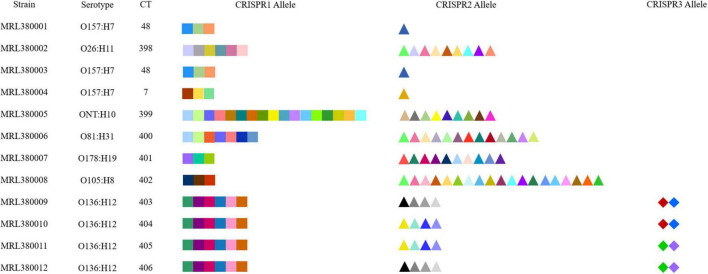
CRISPR spacer arrangements of STEC strains. Elements with different colors and shapes represent different spacers.

CRISPR1, CRISPR2, CRISPR3, and CRISPR4 loci and spacers of STEC screened by our laboratory were identified and extracted, as shown in Table 1. A total of 156 spacers in 12 STEC strains were checked, including 107 unique spacers. Also, 18 unique spacers from 12 STEC strains were not found in the 789 DEC genomes downloaded from the database. Therefore, the CRISPR typing of strains was acquired after spacers of STEC were sorted based on assigning new unique spacers. The spacer arrangement is shown in Figure 3. All 3 strains (MRL380004, MRL380001, and MRL380003) were the same CRISPR typing as the downloaded strains, and two belong to CT48 and one belongs to CT7. In addition, all the strains of CT7 and CT48 were O157:H7 serotype.

**TABLE 1:** CRISPR types and serotypes of laboratory STEC.

Strains	Serotype	CRISPR type	Number of spacers	CRISPR loci
MRL380001	O157:H7	CT48	4	CRISPR1/CRISPR2
MRL380002	O26:H11	CT398	15	CRISPR1/CRISPR2
MRL380003	O157:H7	CT48	4	CRISPR1/CRISPR2
MRL380004	O157:H7	CT7	4	CRISPR1/CRISPR2
MRL380005	ONT:H10	CT399	26	CRISPR1/CRISPR2
MRL380006	O81:H31	CT400	20	CRISPR1/CRISPR2
MRL380007	O178:H19	CT401	13	CRISPR1/CRISPR2
MRL380008	O105:H8	CT402	22	CRISPR1/CRISPR2
MRL380009	O136:H12	CT403	12	CRISPR1/CRISPR2/CRISPR3
MRL380010	O136:H12	CT404	12	CRISPR1/CRISPR2/CRISPR3
MRL380011	O136:H12	CT405	12	CRISPR1/CRISPR2/CRISPR3
MRL380012	O136:H12	CT406	12	CRISPR1/CRISPR2/CRISPR3

The results indicated that the spacers of STEC are relatively diverse. The spacers of CRISPR1 loci of MRL380009, MRL380010, MRL380011, and MRL380012 strains were completely consistent, and the spacer sequences of MRL380009 and MRL380012 strains were consistent at CRISPR2 loci. However, the distribution of spacers at CRISPR3 loci was inconsistent. Similarly, MRL380010 and MRL380011 strains had the same spacer sequences at CRISPR2 loci, while the spacer sequences at CRISPR3 loci were different. MRL380009, MRL380010, MRL380011, and MRL380012 belonged to O136:H12 serotype, but belonged to four different CTs.

### Pathogenic Potential of the Strains

#### Relationship Between Diarrheagenic *Escherichia coli* Virulence Gene Quantity and Clustered Regularly Interspaced Short Palindromic Repeats Typing

The type and number of virulence genes of foodborne pathogenic bacteria represent the pathogenic potential of the strain, and the strain with strong pathogenic factors is often more likely to bring pathogenic risk to human. For example, STEC has caused many foodborne disease outbreaks and public health challenges (Karmali, 2017) mostly from *stx*, the main virulence factor of STEC. In this study, 123 strains were positive for *stx1* only and 237 were positive for *stx2* only. There was difference in CRISPR types between strains carrying only *stx1* or *stx2*. The virulence genes were analyzed and it was found that *stx2* was more prevalent than *stx1* in STEC. In STEC strains isolated from animals, food, and clinical samples by Kruger et al. (2015), *stx2* was more popular, while other studies have shown that the prevalence of *stx1* was higher than that of *stx2* (Wani et al., 2007; Michel and Kase, 2009). These results may be caused by the differences between the isolation place and source.

Typical virulence genes of EAEC strains are *astA*, *pic*, and *aggR*, and these virulence factors are commonly used as important indicators for EAEC isolation and identification (Hebbelstrup Jensen et al., 2017). Researchers have investigated the prevalence of diarrheagenic *Escherichia coli* pathotypes among diarrhea and healthy children up to 5 years of age in Brazil, and found that EAEC was the most common pathological type, indicating that the pathogenicity of EAEC should not be underestimated (Dias et al., 2016). In a Brazilian study that investigated childhood diarrhea, the toxin gene *astA* was associated with acute diarrhea (Pereira et al., 2007). In our study, *astA* was more popular than *pic* and *aggR* in EAEC (*n* = 116); the clinical strains containing *astA* that were downloaded in this study can cause diarrhea (not mentioned whether children are involved), indicating that EAEC with *astA* possibly possessed high potential pathogenicity.

The strains with *eae*^+^ and *bfp*^+^ were marked as typical EPEC (tEPEC), and the strains with *eae*^+^, *bfp*^–^, *stx1*^–^, and *stx2*^–^ were recorded as atypical EPEC (aEPEC) (Spano et al., 2021). Among the EPEC (*n* = 105) downloaded in this study, atypical EPEC covered a relatively high proportion (75.24%), which was similar to the results of other studies (Afset et al., 2004).

A potential contributor to the less reports than other DEC strains, is that EIEC is often observed as a rare cause of diarrhea compared with other DECs (Vieira et al., 2007). However, EIEC is a leading cause of bacterial dysentery with fever, abdominal cramps, diarrhea, and other symptoms in places with poor sanitation (Farajzadeh-Sheikh et al., 2020). Therefore, its pathogenic risk should not be ignored, and it is necessary that the research of the distribution of virulence genes is more conducive to understand the pathogenic potential of bacteria.

Enterotoxigenic *E. coli* is a global diarrhea pathogen that causes disease in mammalian hosts infected by adhesins and secreted enterotoxins (Crofts et al., 2018). ETEC was first recognized as a cause of human illness in the 1960s (Bradley et al., 1971). ETEC is the most common cause of traveler’s diarrhea and rarely found in meat products, and contaminated food and water have been implicated as vehicles for transmission of ETEC infection. Moreover, ETEC has been studied in India, the United States, and other regions (Daniels, 2005; Crofts et al., 2018; Mondal et al., 2022). While the amount of ETEC collected in this study was limited and originated from Bangladesh, the United States, China, Chile, and other countries, it was impossible to evaluate the geographical specificity of strains in each region.

**FIGURE 4:**
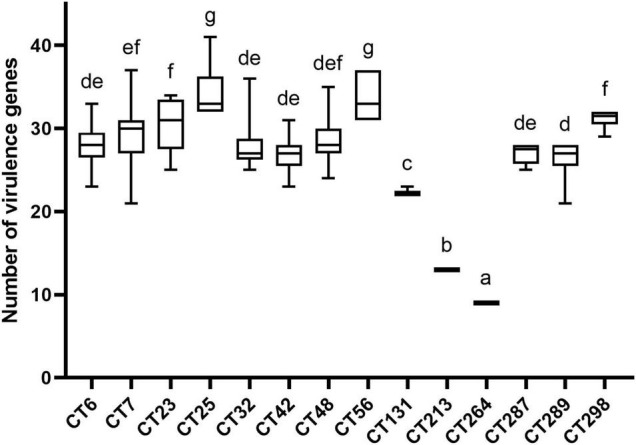
Number of virulence genes among different CRISPR types of DEC strains. The same letter in the figure indicates that there is no significant difference between each other, and the absence of the same letter indicates that there is a significant difference between each other (*p* < 0.01).

Combined with the results of CRISPR typing of DEC strains, there was a significant correlation between CRISPR typing and the number of virulence genes (*p* < 0.01). The number of virulence genes between strains of different CTs was significantly different (*p* < 0.01), indicating that the pathogenicity potential of different CT strains was different. The distribution of virulence gene quantity of DEC strains with different CTs is shown in Figure 4. Virulence genes can not only be used for characteristic identification of strains, but also can make prospective judgments on the potential pathogenic properties of strains. To investigate the virulence potential of different CT strains, we analyzed the distribution of virulence gene quantity in different CTs of DEC strains downloaded (Figure 4). Figure 4 shows the CT of more than ten strains. The strains with the largest number of virulence genes were concentrated in CT25 (H11, H16) and CT56 (H11). CT264 (H14) was the least prevalent followed by CT213 (H21). CT25 and CT56 only had CRISPR1 and CRISPR2 locus, while CT264 had CRISPR3 locus, and the number of spacers in CRISPR1 locus of CT264 is more than that of CT25 and CT56. CT48 (O157:H7) had significant difference with CT25 (H11, H16), CT56 (H11), CT131 (H4), CT213 (H21), and CT264 (H4) (*p* < 0.01), but had no significant difference from other CTs. It can also be found that the number of virulence genes varied with different CRISPR types, and the CRISPR types that were significantly different from other types were H4, H11, H14, and H21 serotypes, indicating that CRISPR typing can distinguish the number of virulence genes of these serotype strains excellently.

**FIGURE 5:**
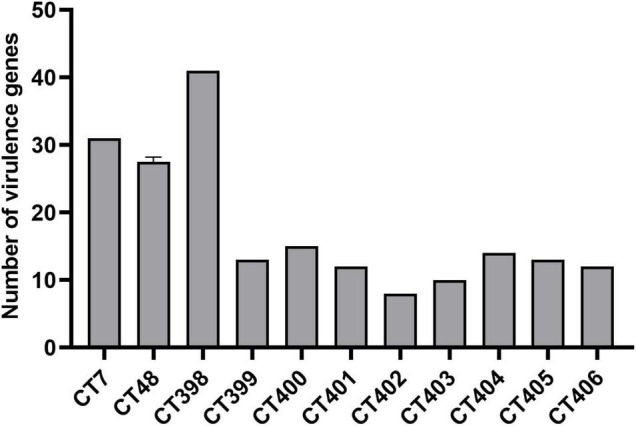
Number of virulence genes among different CRISPR types of laboratory STEC strains.

The distribution of virulence genes in different CRISPR types of laboratory STEC strains is shown in Figure 5. CT398 (O26:H11) was the most prevalent followed by CT7 (O157:H7), and CT402 (O105:H8) had the least number of virulence genes. As the number of strains increases in the future, it is expected to obtain a more accurate relationship between CT and the number of virulence genes.

#### Caco-2 Cell Assay

**FIGURE 6:**
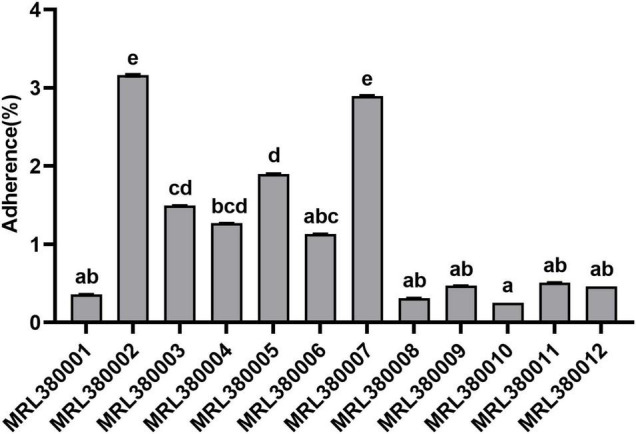
Adherence to of Caco-2 cells by different strains of STEC. The same letter indicates that there was no significant difference between each other, and the absence of the same letter indicates that there was a significant difference between each other (*p* < 0.05). Results were expressed as percent of adherent bacteria with respect to inoculum.

**FIGURE 7:**
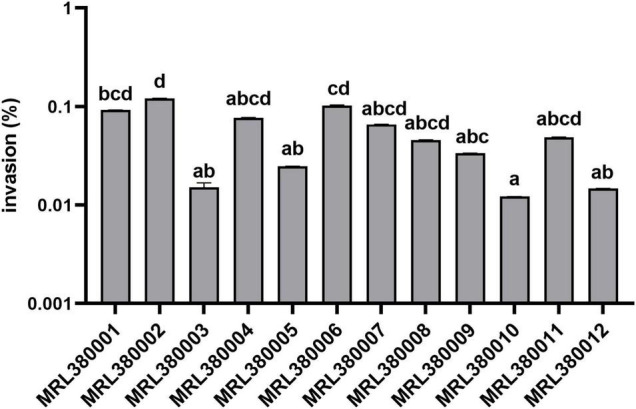
Invasion of Caco-2 cells by different strains of STEC. The same letter indicates that there was no significant difference between each other, and the absence of the same letter indicates that there was a significant difference between each other (*p* < 0.05). Results were expressed as percent of invasive bacteria respect to inoculum.

The virulence potential of the strain is not only reflected in the distribution of virulence genes. To investigate the pathogenic potential of the STEC more comprehensively, the adhesion and invasion capacities of STEC to Caco-2 cells were studied (Figures 6, 7).

MRL380002 and MRL380007 behaved similarly and presented a significantly higher adhesive capacity (*p* < 0.05). MRL380010 had the least adhesive capacity and was not significantly different from that of MRL380001, MRL380006, MRL380008, MRL380009, MRL380010, MRL380011, and MRL380012, while it was significantly lower than MRL380002, MRL380003, MRL380004, MRL380005, and MRL380007 adhesion rate (*p* < 0.05).

The serotype of MRL380002 was O26:H11, which also carried *eae*, *stx1*, and *stx2* virulence genes. The serotype of MRL380007 was O178:H19, which carried *stx2* but did not carry *eae* and *stx1*. MRL380010 had the lowest adhesion rate, and its serotype was O136:H12, which did not carry *eae* and *stx2* genes, but carried *stx1* genes. We found that there were also differences in adhesion ability between strains of the same serotype. The serotypes of MRL380001, MRL380003, and MRL380004 were O157:H7, among which MRL380001 and MRL380003 showed significant differences in adhesion ability to cells. Kobayashi et al. (2016) measured the cell adhesion capacity of 11 STEC strains with serotype O103:H2, and the results showed that there were significant differences in adhesion capacity among strains of the same serotype, which was similar to our results.

MRL380002 had the greatest invasive capacity and it was significantly higher than that of MRL380003, MRL380005, MRL380009, MRL380010, and MRL380012 (*p* < 0.05). The invasion rates of MRL380004, MRL380007, MRL380008, and MRL380011 were not significantly different from other strains. MRL380001, MRL380003, and MRL380004 belonging to O157:H7 showed no significant difference in invasive capacity. Serotypes of MRL380009, MRL380010, MRL380011, and MRL380012 were O136:H12. There was no significant difference in the invasion rate of Caco-2 cells. The adhesion and invasion abilities of MRL380002 were the highest among strains, but the order of adhesion and invasion abilities of other strains was not consistent, indicating that there was not necessarily a correlation between the adhesion ability and invasion ability. In addition, the outstanding adherence to and invasion of Caco-2 epithelial cells by O26:H11 confirmed that the virulence of non-O157 strain might not be underestimated. The serotypes O26:H11 and O103:H2 were used for comparison together with O157:H7 because these serotypes are considered the most important non-O157 STEC serotypes associated with increasing frequency in patients with bloody diarrhea and HUS (Jelacic et al., 2003; Alexander et al., 2005).

**FIGURE 8:**
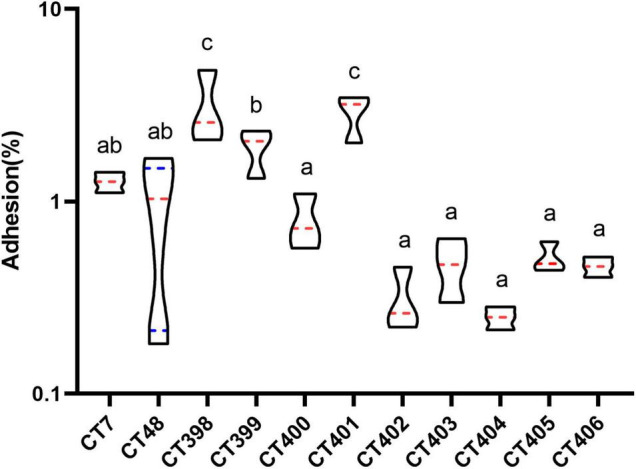
Distribution of adhesive capacity of different CRISPR typing STEC strains. The same letter indicates that there was no significant difference between each other, and the absence of the same letter indicates that there was a significant difference between each other (*p* < 0.05). The red lines represent the median, and the blue lines represent the quartile.

Combined with the CRISPR typing results of STEC, this study also analyzed the differences of adhesion and invasion capacities among strains with different CRISPR type. The distribution of cell adhesion capacity of different CT type STEC strains is shown in Figure 8.

The mean adhesion rate of CT398 was the highest followed by CT401, and adhesion rates of CT398 and CT401 strains to Caco-2 cells were significantly higher than that of other CRISPR types (*p* < 0.05). CT399 adhesion ability was significantly higher than CT400, CT402, CT403, CT404, CT405, and CT406 (*p* < 0.05), but with CT7 and CT48, there was no significant difference between strains. Whether the results of CRISPR typing can predict the adhesive capacity of the strains needs to be further studied by increasing the number of strains.

CT398 had the largest quantity of virulence genes, including *eae* and *stx1*, and CT402 and CT404 with the lowest adhesion rate did not have *eae* because *eae* was a factor related to adhesive capacity (Kobayashi et al., 2016), the lack of *eae* had a certain impact on the adhesive capacity of the strain. Including intimin (*eae*), translocated intimin receptor (*tir*), the type III secretion apparatus (*espB* and *espD*), and homolog adhesion (*iha*) are also associated with adherence to the intestinal epithelium (Tarr et al., 2000; Kobayashi et al., 2016). CT398 with the highest adhesion rate carried *tir* and *espB*. CT7 and CT48 carried *tir* and *espB*, and *iha* had a higher adhesion rate. CT402, CT403, CT404, CT405, CT406 *tir*, *espB*, and *iha* were negative, and their adhesive capacities were inferior. The largest number of spacer sequences were identified in CT402, but CT402 had the lowest number of virulence genes and a low adhesive capacity. How the spacer affects the adhesive capacity of strains is indistinct, and further investigation is needed by increasing the number of strains.

**FIGURE 9:**
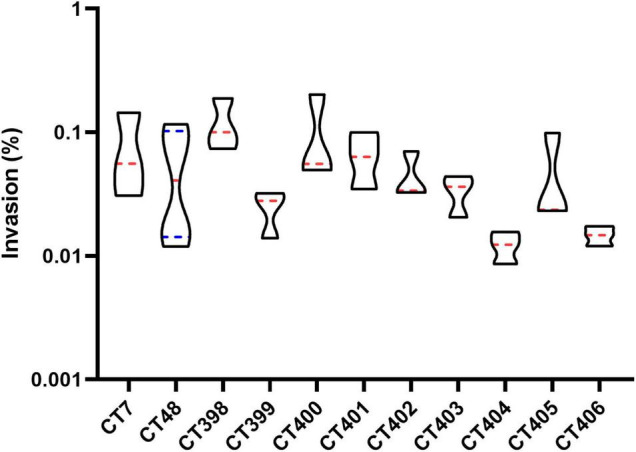
Distribution of invasion capacity of different CRISPR typing STEC strains. The red lines represent the median, and the blue lines represent the quartile.

The invasion capacity of STEC strains of different CRISPR types is shown in Figure 9. CT398 showed the highest invasion value followed by CT400, and CT404 was generally lower than other CT types. CT398 carried *eae* and *stx1*; moreover, the number of virulence genes and adhesion rate are both the highest. CT404 had the least virulence genes quantity and adhesion rate carried *stx1* without *eae*. There was no significant difference in the invasion rate between different CT type strains (*p* > 0.05), which was different from the adhesion capacity.

## Conclusion

In conclusion, the existence rate of CRISPR1 and CRISPR2 loci in DEC strains was generally higher than that of CRISPR3 and CRISPR4 loci. The strains with the same H antigen shared more spacers, while the same O group did not share spacer sequences or shared spacers less. The CRISPR spacers polymorphism showed the potential for DEC typing, providing clues for inferring virulence of strains. The number of virulence genes was different among DEC strains with different CRISPR types, indicating that the pathogenicity potential of CT strains was different. For STEC, the adhesion capacity of different CT strains was significantly different, which provided a basis for CRISPR typing to distinguish the pathogenicity of strains. As a whole, with the increase in the number of DEC strains and the popularization of CRISPR typing method, it is expected to judge the pathogenic potential of the strain by CRISPR type. Moreover, paying attention to CT strains with strong virulence potential in advance is beneficial to reduce the occurrence of food safety problems.

## Data Availability Statement

The original contributions presented in the study are included in the article/Supplementary Material, further inquiries can be directed to the corresponding author/s.

## Author Contributions

QD and HL conceived of the study and modified the first draft of the article. ZB was responsible for the experimental work, article writing, and data analysis. SZ isolated laboratory strains. XW and WW provided help with research ideas. MA modified the article. All authors reviewed and approved the final article.

## Conflict of Interest

The authors declare that the research was conducted in the absence of any commercial or financial relationships that could be construed as a potential conflict of interest.

## Publisher’s Note

All claims expressed in this article are solely those of the authors and do not necessarily represent those of their affiliated organizations, or those of the publisher, the editors and the reviewers. Any product that may be evaluated in this article, or claim that may be made by its manufacturer, is not guaranteed or endorsed by the publisher.

## Footnotes

1
https://cge.cbs.dtu.dk/services/SerotypeFinder/

2
https://cge.cbs.dtu.dk/services/VirulenceFinder/

## References

[B1] Afset J. E. Bevanger L. Romundstad P. Bergh K. (2004). Association of atypical enteropathogenic *Escherichia coli* (EPEC) with prolonged diarrhoea. *J. Med. Microbiol.* 53(Pt 11) 1137–1144. 10.1099/jmm.0.45719-0 15496393

[B2] Alexander M. Martina B. Zimmerhackl L. B. Rita P. Dag H. Helmut T. (2005). Enterohemorrhagic *Escherichia coli* in human infection: *in vivo* evolution of a bacterial pathogen. *Clin. Infect. Dis.* 6 785–792. 10.1086/432722 16107974

[B3] Boerlin P. Mcewen S. A. Boerlin-Petzold F. Wilson J. B. Gyles C. (1999). Associations between virulence factors of Shiga toxin-producing *Escherichia coli* and disease in humans. *J. Clin. Microbiol.* 37 497–503. 10.1128/JCM.37.3.497-503.1999 9986802 PMC84443

[B4] Bolotin A. Quinquis B. Sorokin A. Ehrlich S. D. (2005). Clustered regularly interspaced short palindrome repeats (CRISPRs) have spacers of extrachromosomal origin. *Microbiology* 151(Pt 8) 2551–2561. 10.1099/mic.0.28048-0 16079334

[B5] Bradley S. R. Gorbach S. L. Banwell J. G. Benedicta J. Chatterjee B. D. Mitra R. (1971). Enterotoxigenic *Escherichia coli* isolated from patients with severe cholera-like disease. *J. Infect. Dis.* 123 378–385. 10.1093/infdis/123.4.378 4938945

[B6] Bruyand M. Mariani-Kurkdjian P. Gouali M. de Valk H. King L. A. Le Hello S. (2018). Hemolytic uremic syndrome due to Shiga toxin-producing *Escherichia coli* infection. *Med. Mal. Infect.* 48 167–174. 10.1016/j.medmal.2017.09.012 29054297

[B7] Crofts A. A. Giovanetti S. M. Rubin E. J. Poly F. M. Gutierrez R. L. Talaat K. R. (2018). Enterotoxigenic *E. coli* virulence gene regulation in human infections. *Proc. Natl. Acad. Sci. U.S.A.* 115 E8968–E8976. 10.1073/pnas.1808982115 30126994 PMC6156659

[B8] Croxen M. A. Finlay B. B. (2010). Molecular mechanisms of *Escherichia coli* pathogenicity. *Nat. Rev. Microbiol.* 8 26–38. 10.1038/nrmicro2265 19966814

[B9] Daniels N. A. (2005). Enterotoxigenic *Escherichia coli*: traveler’s diarrhea comes home. *Clin. Infect. Dis.* 42 335–336. 10.1086/499249 16392077

[B10] Deveau H. Garneau J. E. Moineau S. (2010). CRISPR/Cas system and its role in phage-bacteria interactions. *Annu. Rev. Microbiol.* 64 475–493. 10.1146/annurev.micro.112408.134123 20528693

[B11] Dias R. C. Dos Santos B. C. Dos Santos L. F. Vieira M. A. Yamatogi R. S. Mondelli A. L. (2016). Diarrheagenic *Escherichia coli* pathotypes investigation revealed atypical enteropathogenic *E. coli* as putative emerging diarrheal agents in children living in Botucatu. Sao Paulo State, Brazil. *APMIS* 124 299–308. 10.1111/apm.12501 26752102

[B12] Díez-Villaseñor C. Almendros C. García-Martínez J. Mojica F. J. M. (2010). Diversity of CRISPR loci in *Escherichia coli*. *Microbiology* 156 1351–1361. 10.1099/mic.0.036046-0 28206910

[B13] Farajzadeh-Sheikh A. Savari M. Ahmadi K. Hosseini Nave H. Shahin M. Afzali M. (2020). Distribution of genes encoding virulence factors and the genetic diversity of enteroinvasive *Escherichia coli* (EIEC) Isolates from Patients with Diarrhea in Ahvaz, Iran. *Infect. Drug Resist.* 13 119–127. 10.2147/IDR.S235009 32021326 PMC6963944

[B14] Fujioka M. Otomo Y. Ahsan C. R. (2013). A novel single-step multiplex polymerase chain reaction assay for the detection of diarrheagenic *Escherichia coli*. *J. Microbiol. Methods* 92 289–292. 10.1016/j.mimet.2012.12.010 23270615

[B15] Gomes T. A. Elias W. P. Scaletsky I. C. Guth B. E. Rodrigues J. F. Piazza R. M. (2016). Diarrheagenic *Escherichia coli*. *Braz. J. Microbiol.* 47(Suppl. 1) 3–30. 10.1016/j.bjm.2016.10.015 27866935 PMC5156508

[B16] Grissa I. Vergnaud G. Pourcel C. (2007). CRISPRFinder: a web tool to identify clustered regularly interspaced short palindromic repeats. *Nucleic Acids Res.* 35 W52–W57. 10.1093/nar/gkm360 17537822 PMC1933234

[B17] Hebbelstrup Jensen B. Poulsen A. Hebbelstrup Rye Rasmussen S. Struve C. Engberg J. H. Friis-Moller A. (2017). Genetic virulence profile of enteroaggregative *Escherichia coli* strains isolated from danish children with either acute or persistent diarrhea. *Front. Cell Infect. Microbiol.* 7:230. 10.3389/fcimb.2017.00230 28611957 PMC5447714

[B18] Hernandes R. T. Elias W. P. Vieira M. A. Gomes T. A. (2009). An overview of atypical enteropathogenic *Escherichia coli*. *FEMS Microbiol. Lett.* 297 137–149. 10.1111/j.1574-6968.2009.01664.x 19527295

[B19] Hunter P. R. Gaston M. A. (1988). Numerical index of the discriminatory ability of typing systems: an application of Simpson’s index of diversity. *J. Clin. Microbiol.* 26 2465–2466. 10.1128/jcm.26.11.2465-2466.1988 3069867 PMC266921

[B20] Jelacic J. K. Damrow T. Chen G. S. Jelacic S. Bielaszewska M. Ciol M. (2003). Shiga toxin–producing *Escherichia coli* in montana: bacterial genotypes and clinical profiles. *J. Infect. Dis.* 188 719–729. 10.1086/376999 12934188

[B21] Joensen K. G. Tetzschner A. M. Iguchi A. Aarestrup F. M. Scheutz F. (2015). Rapid and easy *in silico* serotyping of *Escherichia coli* isolates by use of whole-genome sequencing data. *J. Clin. Microbiol.* 53 2410–2426. 10.1128/JCM.00008-15 25972421 PMC4508402

[B22] Karmali M. A. (2017). Emerging public health challenges of shiga toxin-producing *escherichia coli* related to changes in the pathogen, the population, and the environment. *Clin. Infect. Dis.* 64 371–376. 10.1093/cid/ciw708 27986670

[B23] Karmali M. A. (2018). Factors in the emergence of serious human infections associated with highly pathogenic strains of shiga toxin-producing *Escherichia coli*. *Int. J. Med. Microbiol.* 308 1067–1072. 10.1016/j.ijmm.2018.08.005 30146439

[B24] Karmali M. Petric M. Steele B. Lim C. J. L. (1983). Sporadic cases of haemolytic-uraemic syndrome associated with faecal cytotoxin and cytotoxin-producing *Escherichia coli* in stools. *Lancet* 321 619–620. 10.1016/s0140-6736(83)91795-6 6131302

[B25] Keir L. S. Marks S. D. Kim J. J. (2012). Shigatoxin-associated hemolytic uremic syndrome: current molecular mechanisms and future therapies. *Drug Des. Dev. Ther.* 6 195–208. 10.2147/DDDT.S25757 22888220 PMC3414372

[B26] Klingberg T. D. Lesnik U. Arneborg N. Raspor P. Jespersen L. (2008). Comparison of Saccharomyces cerevisiae strains of clinical and nonclinical origin by molecular typing and determination of putative virulence traits. *FEMS Yeast Res.* 8 631–640. 10.1111/j.1567-1364.2008.00365.x 18355272 PMC2430332

[B27] Kobayashi N. Maeda E. Saito S. Furukawa I. Hara-Kudo Y. J. (2016). Association of cell-adhesion activities with virulence in shiga toxin-producing *Escherichia coli* O103:H2. *Biocontrol Sci.* 21 57–61. 10.4265/bio.21.57 27009511

[B28] Kruger A. Lucchesi P. M. Sanso A. M. Etcheverria A. I. Bustamante A. V. Burgan J. (2015). Genetic characterization of Shiga toxin-producing *Escherichia coli* O26:H11 strains isolated from animal, food, and clinical samples. *Front. Cell Infect. Microbiol.* 5:74. 10.3389/fcimb.2015.00074 26539413 PMC4612136

[B29] Kyle J. L. Cummings C. A. Parker C. T. Quinones B. Vatta P. Newton E. (2012). *Escherichia coli* serotype O55:H7 diversity supports parallel acquisition of bacteriophage at Shiga toxin phage insertion sites during evolution of the O157:H7 lineage. *J. Bacteriol.* 194 1885–1896. 10.1128/JB.00120-12 22328665 PMC3318487

[B30] Larkin M. A. Blackshields G. Brown N. P. Chenna R. McGettigan P. A. McWilliam H. (2007). Clustal W and Clustal X version 2.0. *Bioinformatics* 23 2947–2948. 10.1093/bioinformatics/btm404 17846036

[B31] Li Q. Wang X. Yin K. Hu Y. Xu H. Xie X. (2018). Genetic analysis and CRISPR typing of *Salmonella enterica* serovar Enteritidis from different sources revealed potential transmission from poultry and pig to human. *Int. J. Food Microbiol.* 266 119–125. 10.1016/j.ijfoodmicro.2017.11.025 29212058

[B32] Long J. Xu Y. Ou L. Yang H. Xi Y. Chen S. (2019). Polymorphism of Type I-F CRISPR/Cas system in *Escherichia coli* of phylogenetic group B2 and its application in genotyping. *Infect. Genet. Evol.* 74:103916. 10.1016/j.meegid.2019.103916 31195154

[B33] Makarova K. S. Haft D. H. Barrangou R. Brouns S. J. Charpentier E. Horvath P. (2011). Evolution and classification of the CRISPR-Cas systems. *Nat. Rev. Microbiol.* 9 467–477. 10.1038/nrmicro2577 21552286 PMC3380444

[B34] Manuel E. Gordillo Gordon R. Reeve Janeene P. Mathewson J. J. Dupont H. L. Murray B. E. (1992). Molecular characterization of strains of enteroinvasive *Escherichia coli* O143, including isolates from a large outbreak in Houston, Texas. *J. Clin. Microbiol.* 30 889–893. 10.1128/jcm.30.4.889-893.1992 1349307 PMC265180

[B35] Marraffini L. A. Sontheimer E. J. (2008). CRISPR interference limits horizontal gene transfer in staphylococci by targeting DNA. *Science* 322 1843–1845. 10.1126/science.1165771 19095942 PMC2695655

[B36] Marraffini L. A. Sontheimer E. J. (2010). CRISPR interference: RNA-directed adaptive immunity in bacteria and archaea. *Nat. Rev. Genet.* 11 181–190. 10.1038/nrg2749 20125085 PMC2928866

[B37] Michel P. A. Kase J. A. (2009). Genetic profiles of Shiga toxin and intimin genes found in stool broth cultures: a 2-year reference laboratory study. *Diagn. Microbiol. Infect. Dis.* 65 85–92. 10.1016/j.diagmicrobio.2009.06.006 19748416

[B38] Mojica F. J. Diez-Villasenor C. Garcia-Martinez J. Soria E. (2005). Intervening sequences of regularly spaced prokaryotic repeats derive from foreign genetic elements. *J. Mol. Evol.* 60 174–182. 10.1007/s00239-004-0046-3 15791728

[B39] Mondal I. Bhakat D. Chowdhury G. Manna A. Samanta S. Deb A. K. (2022). Distribution of virulence factors and its relatedness towards the antimicrobial response of enterotoxigenic *Escherichia coli* strains isolated from patients in Kolkata, India. *J. Appl. Microbiol.* 132 675–686. 10.1111/jam.15206 34242448

[B40] Muniesa M. Jofre J. García-Aljaro C. Blanch A. R. (2006). Occurrence of *Escherichia coli* O157:H7 and other enterohemorrhagic *Escherichia coli* in the environment. *Environ. Sci. Technol.* 40 7141–7149. 10.1021/es060927k 17180960

[B41] Olesen I. Jespersen L. (2010). Relative gene transcription and pathogenicity of enterohemorrhagic *Escherichia coli* after long-term adaptation to acid and salt stress. *Int. J. Food Microbiol.* 141 248–253. 10.1016/j.ijfoodmicro.2010.05.019 20603024

[B42] Pai C. H. Ahmed N. Lior H. Johnson W. M. Sims H. V. Woods D. E. (1988). Epidemiology of sporadic diarrhea due to verocytotoxin-producing *Escherichia coli*: a two-year prospective study. *J. Infect. Dis.* 157 1054–1057. 10.1093/infdis/157.5.1054 3283256

[B43] Pereira A. L. Ferraz L. R. Silva R. S. Giugliano L. G. (2007). Enteroaggregative *Escherichia coli* virulence markers: positive association with distinct clinical characteristics and segregation into 3 enteropathogenic *E. coli* Serogroups. *J. Infect. Dis.* 195 366–374. 10.1086/510538 17205475

[B44] Pourcel C. Salvignol G. Vergnaud G. (2005). CRISPR elements in *Yersinia pestis* acquire new repeats by preferential uptake of bacteriophage DNA, and provide additional tools for evolutionary studies. *Microbiology* 151(Pt 3) 653–663. 10.1099/mic.0.27437-0 15758212

[B45] Riley L. W. Remis R. S. Helgerson S. D. Mcgee H. B. Wells J. G. (1983). Hemorrhagic colitis associated with a rare *Escherichia coli* Serotype. *NEJM* 308 681–685. 10.1056/NEJM198303243081203 6338386

[B46] Rodrigues V. F. V. Rivera I. N. G. Lim K. Y. Jiang S. C. (2016). Detection and risk assessment of diarrheagenic *E. coli* in recreational beaches of Brazil. *Mar. Pollut. Bull.* 109 163–170. 10.1016/j.marpolbul.2016.06.007 27301685

[B47] Rump L. V. Feng P. C. Fischer M. Monday S. R. (2010). Genetic analysis for the lack of expression of the O157 antigen in an O Rough:H7 *Escherichia coli* strain. *Appl. Environ. Microbiol.* 76 945–947. 10.1128/AEM.02046-09 19948859 PMC2813021

[B48] Scaletsky I. C. A. Fabbricotti S. H. Silva S. O. C. Morais M. B. Fagundes-Neto U. (2002). HEp-2–Adherent *Escherichia coli* strains associated with acute infantile diarrhea, São Paulo, Brazil. *Emerg. Infect. Dis.* 8 855–858. 10.3201/eid0808.010492 12141974 PMC2732515

[B49] Shahbazi G. Rezaee M. A. Nikkhahi F. Ebrahimzadeh S. Hemmati F. Namarvar B. B. (2021). Characteristics of diarrheagenic *Escherichia coli* pathotypes among children under the age of 10 years with acute diarrhea. *Gene Rep.* 25:101318. 10.1016/j.genrep.2021.101318

[B50] Shen J. Rump L. Ju W. Shao J. Zhao S. Brown E. (2015). Virulence characterization of non-O157 Shiga toxin-producing *Escherichia coli* isolates from food, humans and animals. *Food Microbiol.* 50 20–27. 10.1016/j.fm.2015.02.007 25998811

[B51] Spano L. C. Guerrieri C. G. Volpini L. P. B. Schuenck R. P. Goulart J. P. Boina E. (2021). EHEC O111:H8 strain and norovirus GII.4 Sydney [P16] causing an outbreak in a daycare center. Brazil, 2019. *BMC Microbiol.* 21:95. 10.1186/s12866-021-02161-x 33781202 PMC8008580

[B52] Tarr P. I. Bilge S. S. Vary J. C. Jelacic S. Habeeb R. L. Ward T. R. (2000). Iha: a novel *Escherichia coli* O157:H7 adherence-conferring molecule encoded on a recently acquired chromosomal island of conserved structure. *Infect. Immun.* 68 1400–1407. 10.1128/IAI.68.3.1400-1407.2000 10678953 PMC97294

[B53] Toro M. Cao G. Ju W. Allard M. Barrangou R. Zhao S. (2014). Association of clustered regularly interspaced short palindromic repeat (CRISPR) elements with specific serotypes and virulence potential of shiga toxin-producing *Escherichia coli*. *Appl. Environ. Microbiol.* 80 1411–1420. 10.1128/AEM.03018-13 24334663 PMC3911044

[B54] Vieira N. Bates S. J. Solberg O. D. Ponce K. Howsmon R. Cevallos W. (2007). High prevalence of enteroinvasive *Escherichia coli* isolated in a remote region of northern coastal Ecuador. *Am. J. Trop. Med. Hyg.* 76 528–533. 10.4269/ajtmh.2007.76.528 17360879 PMC2396511

[B55] Wang Q. Perepelov A. V. Wen L. Shashkov A. S. Wang X. Guo X. (2012). Identification of the two glycosyltransferase genes responsible for the difference between *Escherichia coli* O107 and O117 O-antigens. *Glycobiology* 22 281–287. 10.1093/glycob/cwr137 21968437

[B56] Wani S. A. Hussain I. Nabi A. Fayaz I. Nishikawa Y. (2007). Variants of eae and stx genes of atypical enteropathogenic *Escherichia coli* and non-O157 Shiga toxin-producing *Escherichia coli* from calves. *Lett. Appl. Microbiol.* 45 610–615. 10.1111/j.1472-765X.2007.02235.x 17916128

[B57] Xiong Y. Wang P. Lan R. Ye C. Wang H. (2012). A Novel *Escherichia coli* O157:H7 clone causing a major hemolytic uremic syndrome outbreak inChina. *PLoS One* 7:e36144. 10.1371/journal.pone.0036144 22558360 PMC3338595

[B58] Yan X. Fratamico P. Bono J. Baranzoni G. Chen C. J. (2015). Genome sequencing and comparative genomics provides insights on the evolutionary dynamics and pathogenic potential of different H-serotypes of Shiga toxin-producing *Escherichia coli* O104. *BMC Microbiol.* 15:83. 10.1186/s12866-015-0413-9 25887577 PMC4393859

[B59] Yeh H. Y. Awad A. (2020). Genotyping of *Campylobacter jejuni* isolates from poultry by clustered regularly interspaced short palindromic repeats (CRISPR). *Curr. Microbiol.* 77 1647–1652. 10.1007/s00284-020-01965-w 32279188

[B60] Yin S. Jensen M. A. Bai J. Debroy C. Barrangou R. Dudley E. G. (2013). The evolutionary divergence of Shiga toxin-producing *Escherichia coli* is reflected in clustered regularly interspaced short palindromic repeat (CRISPR) spacer composition. *Appl. Environ. Microbiol.* 79 5710–5720. 10.1128/AEM.00950-13 23851088 PMC3754162

[B61] Zeng H. Li C. He W. Zhang J. Chen M. Lei T. (2019). *Cronobacter sakazakii, Cronobacter malonaticus, and Cronobacter dublinensis* genotyping based on CRISPR locus diversity. *Front. Microbiol.* 10:1989. 10.3389/fmicb.2019.01989 31555228 PMC6722223

